# Disparities in survival by stage after surgery between pancreatic head and body/tail in patients with nonmetastatic pancreatic cancer

**DOI:** 10.1371/journal.pone.0226726

**Published:** 2019-12-19

**Authors:** Zhenjiang Zheng, Mojin Wang, Chunlu Tan, Yonghua Chen, Jie Ping, Rui Wang, Xubao Liu

**Affiliations:** 1 Department of Pancreatic Surgery, West China Hospital, Sichuan University, Chengdu, Sichuan Province, China; 2 Department of Gastrointestinal Surgery, Institute of Digestive Surgery and State Key Laboratory of Biotherapy, West China Hospital, Sichuan University, Chengdu, Sichuan Province, China; 3 Division of Epidemiology, Vanderbilt University Medical Center, Nashville, TN, United States of America; 4 Department of Gastroenterology, West China Hospital, Sichuan University, Chengdu, Sichuan Province, China; VA Boston Healthcare System, Harvard Medical School (Brigham and Women's Hospital), UNITED STATES

## Abstract

**Background:**

The survival of pancreatic cancer patients with lesions in different locations is unclear. In addition, the different surgery types for nonmetastatic pancreatic head cancer (PHC) or body/tail cancer (PBTC) have different prognostic influences. We analyzed the association by stage between tumor location (head vs. body/tail) and survival of nonmetastatic pancreatic cancer patients who underwent surgery.

**Methods:**

We identified stages I to III pancreatic cancer patients who underwent surgery from 2004 through 2015 by using the Surveillance, Epidemiology, and End Results (SEER) database. The adjusted hazard ratio (HR) and 95% confidence interval (CI) for cancer-specific survival (CSS) were obtained using Cox regression.

**Results:**

A total of 13517 patients or 86.6% had PHC. PHC patients were more likely to have an advanced tumor stage, higher tumor grade, and more frequent and a higher number of positive lymph nodes compared with PBTC patients. The PHC patients had a worse CSS than PBTC patients (P<0.001) and were predominantly at stage I (P = 0.008) and II (P = 0.004). Multivariate Cox regression analysis showed that PHC was an independent prognostic factor associated with a worse CSS in pancreatic cancer patients (HR 1.132, 95% CI 1.042–1.228, P = 0.003), predominantly at stage II (HR 1.128, 95% CI 1.030–1.235, P = 0.009).

**Conclusion:**

At a resectable early stage, the PHC patients had a worse CSS than PBTC patients after surgery. PHC was an independent prognostic factor associated with worse survival in pancreatic cancer patients, predominantly at stage II.

## Introduction

Pancreatic cancer is the fourth leading cause of cancer-related deaths for both men and women in the United States, with 56770 new cases and 45750 deaths in 2019. [1] Pancreatic cancer can be divided into head and body/tail cancer according to the anatomy location. The incidence rate for pancreatic head cancer (PHC) has remained at 5.6% per 100000, whereas the rate for pancreatic body/tail cancers (PBTC) has increased by 46% between 1973 and 2002. [2] Some studies have demonstrated that PHC and PBTC present with different clinical symptoms. [3, 4] Patients with a lack of obvious symptoms, such as obstructive jaundice and PBTC patients, tend to be diagnosed at a more advanced stage than PHC. [2, 3] This is a possibly why of all patients, PBTC patients have a worse survival compared with PHC. [2, 3, 5] Interestingly, for patients at the local stage, PBTC has better survival than PHC. [2]

With regard to the patients who have undergone tumor resection, there have been conflicting results regarding the relationship between tumor location and prognosis (Table 1). Artinyan et al.[5] reported, using the Surveillance, Epidemiology, and End Results (SEER) database, that tumor location was not associated with survival in an unadjusted analysis. In the adjusted analysis, PBTC was a significant prognostic factor for worse survival. However, this study was not conducted during a recent study period (1988–2004). In 2018, by using another population-based database in the Netherlands, van Erning et al.[6] demonstrated that median overall survival was similar between different tumor locations. The result was consistent with previous single-institution studies. [7–9] However, in the population-based study, 4% of patients with pancreatic tail cancer had an inappropriate surgical procedure (e.g., pancreaticoduodenectomy). In addition, single-institution studies have been limited by small sample sizes. More recently, Winer et al.[10] found, by using the National Cancer Database, that patients with PHC had worse overall survival than either body or tail locations for all stages combined. However, the surgical procedures were unclear. Furthermore, no study to date has revealed the diversity by stage.

**Table 1:** Studies discussing the prognostic relevance of the tumor location.

Study(years)	Study interval	Country	Study type	No. of patients		median OS (months)		P
				head	body/tail	head	body/tail	
Artinyan et al.(2008)[5]^a^	1988–2004	America	Population-based	5118	663	NR	NR	-
van Erning et al.(2018)[6]	2005–2015	Netherlands	Population-based	2311	104/206*	16.8	15.0/17.3*	0.156
Ruess et al.(2015)[7]	1994–2014	Germany	Single center	336	61	20.4	24.4	0.284
Toomey et al.(2012)[8]	1991–2009	America	Single center	220	33	16.8	15.2	0.34
Wade et al.(1995)[9]	1987–1991	America	Population-based	252	29	453**	646**	NS
Winer et al.(2019)[10]	1998–2011	America	Population-based	32990	2912/5078*	19.9	26.7/32.8*	<0.001

^a^ The association between tumor location and prognosis was evaluated by Cox proportional hazards regression analysis. By univariate analysis, HR body/tail versus head 1.09 (95% CI 0.99–1.19). By multivariate analysis, HR body/tail versus head 1.11 (95% CI 1.00–1.23).

*Tumor location of body and tail were analyzed separately.

** Values were expressed as mean. The unit is days.

OS, overall survival; NR, Not reported; NS, Not significant; HR, hazard ratio; CI, confidence interval

The objective of the present study was to investigate the impact of tumor location on cancer-specific survival (CSS) for patients who underwent curative intent surgery. Furthermore, we also examined whether this relationship is consistent across tumor stages.

## Materials and methods

### Data source and patient selection

The data were obtained from the SEER program of the National Cancer Institute. The SEER program provides cancer incidence and survival information from 18 population-based registries covering approximately 34.6% of the United States population. Data on patient demographics, years of diagnosis, tumor location, histologic type, tumor stage, tumor grade, cause of death, and treatment were extracted by permitted SEER ID 10457-Nov2017. This study did not require ethical consent, as the SEER data were analyzed anonymously and were publicly available.

Patients with a diagnosis of pancreatic cancer from 2004 through 2015 were extracted. The primary cancer site was identified by the International Classification of Diseases for Oncology, Third Edition (ICD-O-3) codes. The site codes used included the following: pancreatic head (C25.0), pancreatic body (C25.1), and pancreatic tail (C25.2). Behavior item 3 was used to determine the malignant tumor. ICD-O-3 histology codes 8000, 8010, 8140, 8141, 8144, 8211, 8255, 8480, 8481, 8490, 8500, and 8503 were used to identify pancreatic adenocarcinoma. The inclusion criteria were as follows: (1) age at diagnosis was more than 18 years old; (2) diagnosis was microscopically confirmed; and (3) patients received curative intent surgery (surgery of primary site codes: 30, 35, 36, 37, 40, 60, 70). Patients with discordant procedure and site codes (e.g., pancreaticoduodenectomy for PBTC or distal pancreatectomy for PHC) were excluded, as were patients with metastatic disease.

### Data analysis

PHC patients who underwent pancreaticoduodenectomy (codes: 35–37) or total pancreatectomy (codes: 40 and 60) were compared with PBTC patients who underwent distal pancreatectomy (code: 30) or total pancreatectomy (codes: 40 and 60). The primary outcome measure was the CSS, defined as time from diagnosis to death from pancreatic cancer, last date known to be alive, or until last follow-up (November 2017). Age at diagnosis was divided into two groups (70 years or younger and older than 70 years). The number of examined nodes was divided into two groups (15 nodes or less and more than 15 nodes). [11] The number of positive nodes was calculated among patients with node-positive disease. All TNM classifications were restaged according to the criteria described in the American Joint Committee on Cancer (AJCC) Cancer Staging Manual, 7th edition, 2010.

### Statistical analysis

Continuous data were expressed as means and standard deviations. Proportions were presented as numbers and percentages. We compared the clinicopathological characteristics among different tumor locations and stages using the chi-square test for categorical variables and Student's t-test for continuous variables. We utilized the Kaplan-Meier method and log-rank testing for survival analysis. Univariate and multivariate Cox regression analyses were used to evaluate the relationship between tumor location and CSS. According to previous studies and background medical knowledge, we selected several prognostic factors for the Cox proportional model, such as age, sex, race, tumor location, tumor stage, grade, number of examined nodes, radiotherapy, chemotherapy and years of diagnosis. Hazard ratios (HR) were presented with 95% confidence intervals (CI). Two-sided P values at P <0.05 were considered statistically significant. Data were analyzed using SPSS Statistics 22.0 (IBM-SPSS, Chicago, IL).

## Results

### Patient characteristics according to tumor location

A total of 13517 patients (age of diagnosis >18 years) who underwent curative intent surgery for pancreatic cancer from 2004 through 2015 were included in our studies. Among them, 11704 (86.6%) patients had PHC, and 1813 (13.4%) patients had PBTC. Patient characteristics are shown in Table 2. The majority of patients with PHC were male and young patients. There were more White patients with PHC and more Black and other patients with PBTC. Patients with PHC were more likely to have a tumor size smaller than 2 cm, advanced tumor stage, higher T stage, more nodal metastasis (N1), and higher grade. Moreover, PHC patients had a higher frequency of more than 15 examined nodes and more positive nodes. Receiving of adjuvant therapy (radiotherapy and/or chemotherapy) was more prevalent in patients with PHC. The proportion of patients with PBTC increased faster from 2004–2007 to 2012–2015 compared to those with PHC.

**Table 2:** Demographic and clinicopathological features of patients who underwent curative intent surgery for pancreatic cancer.

	Head (n = 11704)	Body/Tail (n = 1813)	P
Sex			<0.001
Male	6042(51.6%)	842(46.4%)	
Female	5662(48.4%)	971(53.6%)	
Age(years)	66.2±10.4	67.4±11.1	<0.001
Race			<0.001
White	9762(83.6%)	1445(79.8%)	
Black	1109(9.5%)	199(11.0%)	
Other	810(6.9%)	167(9.2%)	
Unknown	23	2	
Tumor size(cm)			0.043
≤2	1998(17.5%)	276(15.5%)	
>2	9424(82.5%)	1500(84.5%)	
Unknown	282	37	
T stage			<0.001
T1	616(5.3%)	165(9.2%)	
T2	1310(11.3%)	373(20.8%)	
T3	9131(78.8%)	1171(65.4%)	
T4	535(4.6%)	82(4.6%)	
Unknown	112	22	
N stage			<0.001
N0	3773(32.5%)	925(51.5%)	
N1	7844(67.5%)	872(48.5%)	
Unknown	87	16	
AJCC stage			<0.001
I	1047(9.1%)	391(22.0%)	
II	9938(86.3%)	1309(73.5%)	
III	528(4.6%)	81(4.5%)	
Unknown	191	32	
Grade			<0.001
G1	1143(10.7%)	234(14.3%)	
G2	5480(51.5%)	858(52.6%)	
G3	3920(36.8%)	518(31.7%)	
G4	96(0.9%)	22(1.3%)	
Unknown	1065	181	
Number of examined nodes			<0.001
≤15	6333(54.6%)	1198(66.7%)	
>15	5272(45.4%)	597(33.3%)	
Unknown	99	18	
Number of positive nodes	3.8±3.3	2.8±2.7	<0.001
Any adjuvant therapy			<0.001
Yes	8213(70.5%)	1165(64.8%)	
No	3437(29.5%)	632(35.2%)	
Unknown	54	16	
Years of diagnosis			0.02
2004–2007	3170(27.1%)	439(24.2%)	
2008–2011	4050(34.6%)	630(34.7%)	
2012–2015	4484(38.3%)	744(41.0%)	

### Patient characteristics according to tumor stage and location

Excluding 223 patients who had an unknown tumor stage, the remaining 13294 patients were divided into 3 groups according to tumor stage. The details of the demographic and clinical characteristics of patients at different tumor stages were compared between different tumor locations (Table 3). When comparing PHC to PBTC, there were no significant differences in age distribution, race distribution, tumor size, receiving of adjuvant therapy, and years of diagnosis in the stage I and III subgroups. However, these variables were significantly different in the stage II subgroup. Unlike stage II and III subgroups, PHC patients had a similar number of examined nodes compared to those with PBTC in the stage I subgroup.

**Table 3:** Demographic and clinicopathological features of patients who underwent curative intent surgery for stage I to III pancreatic cancer by stage and location.

	Stage I			Stage II			Stage III		
	Head (n = 1047)	Body/Tail (n = 391)	P	Head (n = 9938)	Body/Tail (n = 1309)	P	Head (n = 528)	Body/Tail (n = 81)	P
Sex			0.024			0.001			0.879
Male	536(51.2%)	174 (44.5%)		5128(51.6%)	611(46.7%)		269(50.9%)	42(51.9%)	
Female	511(48.8%)	217(55.5%)		4810(48.4%)	698(53.3%)		259(49.1%)	39(48.1%)	
Age(years)			0.520			<0.001			0.892
≤70	656(62.7%)	223(57.0%)		6324(63.6%)	743(56.8%)		356(67.4%)	54(66.7%)	
>70	391(37.3%)	168(43.0%)		3614(36.4%)	566(43.2%)		172(32.6%)	27(33.3%)	
Race			0.136			0.002			0.608
White	838(80.4%)	307(78.7%)		8345(84.1%)	1051(80.4%)		427(81.0%)	63(77.8%)	
Black	120(11.5%)	39(10.0%)		906(9.1%)	145(11.1%)		64(12.1%)	10(12.3%)	
Other	84(8.1%)	44(11.3%)		671(6.8%)	112(8.6%)		36(6.8%)	8(9.9%)	
Unknown	5	1		16	1		1	0	
Tumor size(cm)			0.226			<0.001			0.477
≤2	425(40.6%)	145(37.1%)		1504(15.4%)	123(9.5%)		52(10.5%)	6(7.9%)	
>2	622(59.4%)	246(62.9%)		8284(84.6%)	1172(90.5%)		441(89.5%)	70(92.1%)	
Unknown	0	0		150	14		35	5	
T stage			0.226			0.001			N/A
T1	425(40.6%)	145(37.1%)		187(1.9%)	19(1.5%)		0	0	
T2	622(59.4%)	246(62.9%)		676(6.8%)	126(9.6%)		0	0	
T3	0	0		9075(91.3%)	1164(88.9%)		0	0	
T4	0	0		0	0		528(100%)	81(100%)	
Unknown	0	0		0	0		0	0	
N stage			N/A			<0.001			0.002
N0	1047(100%)	391(100%)		2515(25.3%)	487(37.3%)		167(31.8%)	40(49.4%)	
N1	0	0		7414(74.7%)	820(62.7%)		358(68.2%)	41(50.6%)	
Unknown	0	0		9	1		3	0	
Grade			0.029			0.047			0.148
G1	180(20.4%)	86(26.1%)		896(9.7%)	135(11.0%)		52(12.4%)	10(15.2%)	
G2	467(52.8%)	161(48.9%)		4746(51.4%)	659(53.9%)		215(51.3%)	29(43.9%)	
G3	230(26.0%)	75(22.8%)		3500(37.9%)	415(34.0%)		150(35.8%)	25(37.9%)	
G4	7(0.8%)	7(2.1%)		87(0.9%)	13(1.1%)		2(0.5%)	2(3.0%)	
Unknown	163	62		709	87		109	15	
Number of examined nodes			0.101			<0.001			0.013
≤15	737(71.3%)	292(75.6%)		5180(52.4%)	827(63.4%)		301(57.4%)	57(72.2%)	
>15	297(28.7%)	94(24.4%)		4704(47.6%)	477(36.6%)		223(42.6%)	22(27.8%)	
Unknown	13	5		54	5		4	2	
Number of positive nodes	0	0	N/A	3.8±3.3	2.8±2.7	<0.001	3.8±3.3	2.5±1.5	0.032
Any adjuvant therapy			0.051			0.036			0.379
Yes	587(56.3%)	197(50.5%)		7114(71.9%)	895(69.1%)		411(78.4%)	60(74.1%)	
No	456(43.7%)	193(49.5%)		2779(28.1%)	400(30.9%)		113(21.6%)	21(25.94%)	
Unknown	4	1		45	14		4	0	
Years of diagnosis			0.097			0.009			0.881
2004–2007	326(31.1%)	99(25.3%)		2620(26.4%)	304(23.2%)		137(25.9%)	23(28.4%)	
2008–2011	375(35.8%)	150(38.4%)		3444(34.7%)	442(33.8%)		170(32.2%)	26(32.1%)	
2012–2015	346(33.0%)	142(36.3%)		3874(39.0%)	563(43.0%)		221(41.9%)	32(39.5%)	

### Impact of tumor location on CSS at all stages

**Fig 1:**
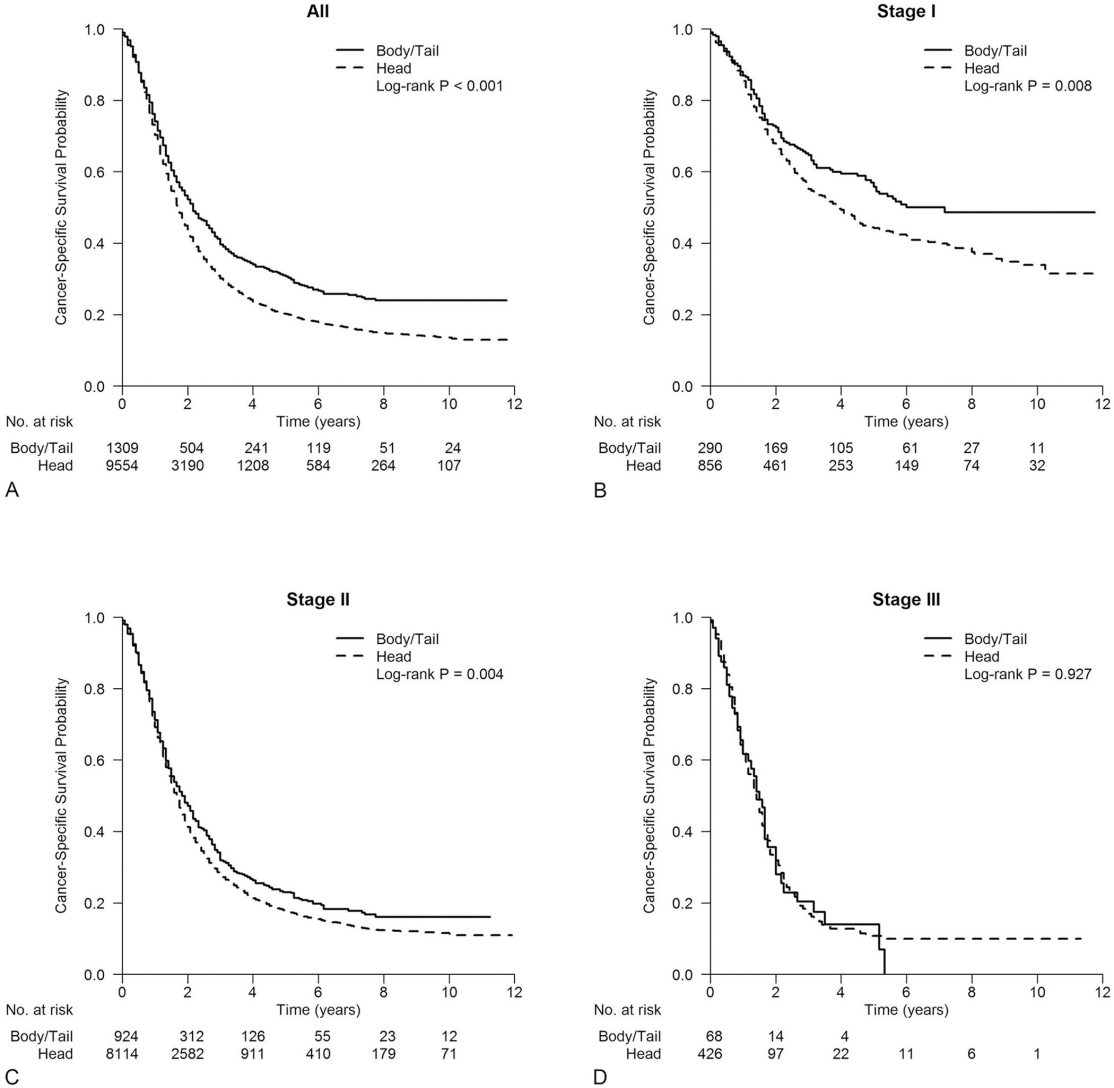
Kaplan-Meier survival analysis for patients with PHC and PBTC. A: All stages combined (P <0.001); B: stage I (P = 0.008); C: stage II (P = 0.004); D: stage III (P = 0.927).

The median follow-up period was 16 months (range, 0–143 months). Kaplan-Meier survival curves for different tumor locations are shown in Fig 1. Overall, as shown in Fig 1A, the median CSS time was 21 months in patients with PHC and 26 months in patients with PBTC (P<0.001). The CSS rates at 1, 3, and 5 years were 70.3%, 30.0% and 20.2% for PHC patients and 74.0%, 39.6% and 30.7% for PBTC patients, respectively. The univariate and multivariate Cox regression analyses for CSS were performed for all stages combined (Table 4). In the univariate Cox regression analysis, PHC, male sex, age older than 70 years, tumor size more than 2 cm, higher T stage, more nodal metastasis (N1), higher AJCC stage, higher grade, number of examined nodes less than 15, no adjuvant therapy (radiotherapy and/or chemotherapy), and years of diagnosis (i.e., 2004–2007, 2008–2011) were predictors of worse CSS. No effect was observed for race distribution. In the multivariate Cox regression analysis, most of these identified poor prognostic features remained independent prognostic factors, with the exception of years of diagnosis from 2008 to 2011.

**Table 4:** Univariate and multivariate analysis of CSS in patients who underwent curative intent surgery for pancreatic cancer.

	Univariate			Multivariate		
	HR	95% CI	P	HR	95% CI	P
Location						
Head	1.283	1.189–1.385	<0.001	1.132	1.042–1.228	0.003
Body/Tail	1			1		
Sex						
Male	1.059	1.010–1.110	0.017	1.092	1.039–1.148	0.001
Female	1			1		
Age						
≤70	0.792	0.754–0.832	<0.001	0.847	0.803–0.894	<0.001
>70	1			1		
Race						
White	1.082	0.987–1.186	0.091	1.002	0.910–1.103	0.965
Black	1.069	0.952–1.199	0.258	1.075	0.952–1.214	0.245
Other	1			1		
Tumor size (cm)						
≤2	0.625	0.584–0.668	<0.001	0.692	0.644–0.743	<0.001
>2	1			1		
T stage						
T1+T2	0.618	0.580–0.659	<0.001	0.830	0.757–0.909	<0.001
T3+T4	1			1		
N stage						
N0	0.561	0.532–0.591	<0.001	0.623	0.584–0.664	<0.001
N1	1			1		
AJCC stage						
I	0.343	0.299–0.393	<0.001	0.526	0.436–0.634	<0.001
II	0.767	0.688–0.854	<0.001	0.693	0.612–0.784	<0.001
III	1			1		
Grade						
G1+G2	0.695	0.662–0.731	<0.001	0.720	0.684–0.757	<0.001
G3+G4	1			1		
Number of examined nodes						
≤15	1.130	1.077–1.185	<0.001	1.226	1.164–1.292	<0.001
>15	1			1		
Any adjuvant therapy						
Yes	1			1		
No	1.474	1.401–1.551	<0.001	1.780	1.683–1.883	<0.001
Years of diagnosis						
2004–2007	1.233	1.157–1.313	<0.001	1.144	1.067–1.225	<0.001
2008–2011	1.096	1.031–1.164	0.003	1.059	0.993–1.130	0.083
2012–2015	1			1		

HR, hazard ratio; CI, confidence interval

### Impact of tumor location on CSS by stage

The CSS of patients at different tumor stages were compared between different tumor locations (Fig 1B–1D). It demonstrated that patients with PHC had a worse CSS for the stage I (P = 0.008) and II subgroups (P = 0.004), but no significant difference was observed in the stage III subgroup (P = 0.927). The similarity in survival persisted in univariate Cox regression analysis (Table 5). The multivariate Cox regression model with tumor location as the explanatory variable showed that PHC was still an independent prognostic factor for worse CSS in the stage II subgroup (HR 1.128, 95% CI 1.030–1.235, P = 0.009). Tumor location was not independently associated with CSS in stage I or III subgroups (HR 1.215, 95% CI 0.970–1.523, P = 0.090; HR 0.887, 95% CI 0.611–1.287, P = 0.528, respectively).

**Table 5:** Univariate and multivariate analysis of CSS in patients who underwent curative intent surgery for pancreatic cancer by stage.

	Stage I			Stage II			Stage III		
	HR	95% CI	P	HR	95% CI	P	HR	95% CI	P
Univariate analysis									
Head	1.320	1.072–1.627	0.009	1.134	1.040–1.236	0.004	0.986	0.721–1.348	0.928
Body/Tail	1			1			1		
Multivariate analysis*									
Head	1.215	0.970–1.523	0.090	1.128	1.030–1.235	0.009	0.887	0.611–1.287	0.528
Body/Tail	1			1			1		

*Cox regression model controlling for sex, age, race, tumor size, tumor grade, T stage, N stage, number of examined nodes, any adjuvant therapy, and years of diagnosis.

HR, hazard ratio; CI, confidence interval

## Discussion

Some studies have demonstrated several differences between PHC and PBTC regarding epidemiology, clinical symptoms, clinicopathologic features, and tumor biology. [2, 3, 6, 7, 12, 13] The only hope of long-term survival for patients with pancreatic cancer is radical surgical resection. However, it is unclear whether these differences could impact prognosis in patients who undergo curative intent surgery. To address this issue in the present study, we included 13517 pancreatic cancer patients from the national data of the SEER program, avoiding the biases associated with single-institution experiences or limited sample sizes. Furthermore, this is the first study to assess the disparity in clinicopathologic features and survival by stage between PHC and PBTC.

Due to a lack of early symptoms, such as obstructive jaundice, PBTC is more advanced at diagnosis than PHC. This is a possible reason why PBTC patients have a worse survival. [2, 3, 5] However, these studies evaluated prognosis among all patients, including those with unresectable tumors. Our study investigated patients who underwent curative intent surgery. PHC patients with tumor resection were more likely to be male, to be younger, to more often have small tumors, and to be diagnosed at a higher frequency of T3/T4 stage compared to PBTC. [5, 10, 13] Similar to our study, we found that PHC also had small tumors but exhibited advanced T stage and more frequent and a higher number of positive nodes. The differences mainly persisted at stage II. It is possible that PHC has more aggressive tumor biology than PBTC at this stage. [12]

In previous studies,[5–10, 12–15] the association between tumor location and prognosis was controversial for patients with tumor resection. By analyzing the data of 6443 patients who underwent cancer-directed surgery in the SEER database, Artinyan et al.[5] demonstrated that PHC and PBTC had a similar prognosis for all stages on univariate analysis. However, when the analysis was controlled for age, positive lymph node status, and T stage, they found that PBTC had a worse overall survival than PHC. These findings conflicted with our results. Potential reasons for this difference are as follows: first, we limited our investigation of patients to those with resectable nonmetastatic pancreatic cancer (stage I to III) who underwent curative intent surgery (i.e., pancreaticoduodenectomy, distal pancreatectomy, and total pancreatectomy) to avoid the biases associated with different treatments and inappropriate surgical procedures. Artinyan et al.[5] additionally included patients who underwent less specific pancreatic resections (e.g., local excision and pancreatectomy). Second, in multivariate analysis, we considered almost all of the factors extracted from the SEER database related to survival. Artinyan et al.[5] only selected tumor location, age, positive lymph node status, and T stage for multivariate analysis. Interestingly, they discovered that the effect of tumor location was lost once the number of examined nodes was added into the multivariate model. [5] Finally, we analyzed more recent data from 2004 through 2015 compared to the study by Artinyan and colleagues (1988–2004). [5] In 2003, Strasberg et al.[16] described a modified technique of distal pancreatectomy named radical antegrade modular pancreatosplenectomy, which provided a greater number of retrieved lymph nodes and a higher percentage of R0 resection compared with standard retrograde pancreatosplenectomy. [17] This has probably contributed to improved prognosis after distal pancreatectomy in recent years. [18, 19]

In this population-based study, we found that patients who underwent curative intent surgery for PHC had a worse CSS than PBTC patients at all stages combined. The result was similar to the study by Winer et al.[10] Although a larger tumor size and fewer examined nodes were observed, PBTC had other pathologically favorable features, with a tendency toward lower AJCC stage, T stage, differentiation, and fewer positive lymph nodes. These factors were identified as independent predictors for survival in our study and in other studies. [5, 10, 13] This may be the explanation for a worse survival in patients with PHC. Furthermore, PHC is an independent prognostic factor for worse survival in multivariate analysis.

Further analysis compared survival by stage between PHC and PBTC. It showed that PHC patients had a worse CSS than PBTC in the stage I and II subgroups, but no significant difference was observed at stage III. For patients at stage I, when we controlled for sex, age, race, tumor grade, T stage, number of examined nodes, any adjuvant therapy, and years of diagnosis, we found that tumor location was not independently associated with CSS. This suggests that a worse CSS at stage I PHC patients is more likely related to other different clinicopathological features between PHC and PBTC. As demonstrated in our study, the majority of PHC patients were male and had a higher tumor grade. For patients at stage II, multivariate Cox regression models showed that PHC was an independent prognostic factor associated with worse CSS. It is speculated that the reasons for worse survival in PHC may be not only different clinicopathological features but also molecular diversity between PHC and PBTC. As reported by Ling et al.[12], PBTC had a less aggressive phenotype associated with lower expression of miR-501-3p compared with PHC at a resectable early stage. In addition, the survival difference in the stage II patients might be due to the fact that it’s easier to get a true negative margin in a successful tail resection than in the head, where the surgeon is constrained by the many vascular structures in place.[10] This idea is indirectly supported by the patterns of recurrence, where even patients with pathological R0 resections often have local recurrences in the resection bed following pancreaticoduodenectomy.[20] Although not statistically significant, our results demonstrated a trend toward better survival among PHC patients at stage III. This is similar to other studies. In a propensity score analysis, Wang et al.[21] found that tumor location was not associated with CSS in patients with stage III disease.

Our study had several limitations. First, information regarding margin status, vascular resections, multivisceral resections and detailed regimens of chemoradiotherapy could not be acquired from the SEER data. It has been reported that tumor location may affect the choice, duration, and response to adjuvant chemotherapy. [22] Second, information on molecular markers was unavailable in the SEER database. We failed to investigate the impact of different tumor biology on the survival of PHC and PBTC. Finally, the models clearly did not adequately account for all variables associated with survival. However, the SEER database includes a large simple size of patients with pancreatic cancer that permits comparison of outcomes across the United States.

In conclusion, at the resectable early stage, the PHC patients had a worse CSS than the PBTC patients after surgery. PHC was an independent prognostic factor associated with worse survival in pancreatic cancer, prominently at stage II. Further study is needed to elucidate the underlying mechanisms for these disparities.
